# Supplementary material for: CA1 and CA3 differentially support spontaneous retrieval of episodic contexts within human hippocampal subfields
Source: Nat Commun. 2018 Jan 18;9:294. doi: 10.1038/s41467-017-02752-1 (PMC5773497; doi:10.1038/s41467-017-02752-1)
Supplement: Supplementary file 1 — Supplementary Information [file 41467_2017_2752_MOESM1_ESM.pdf]

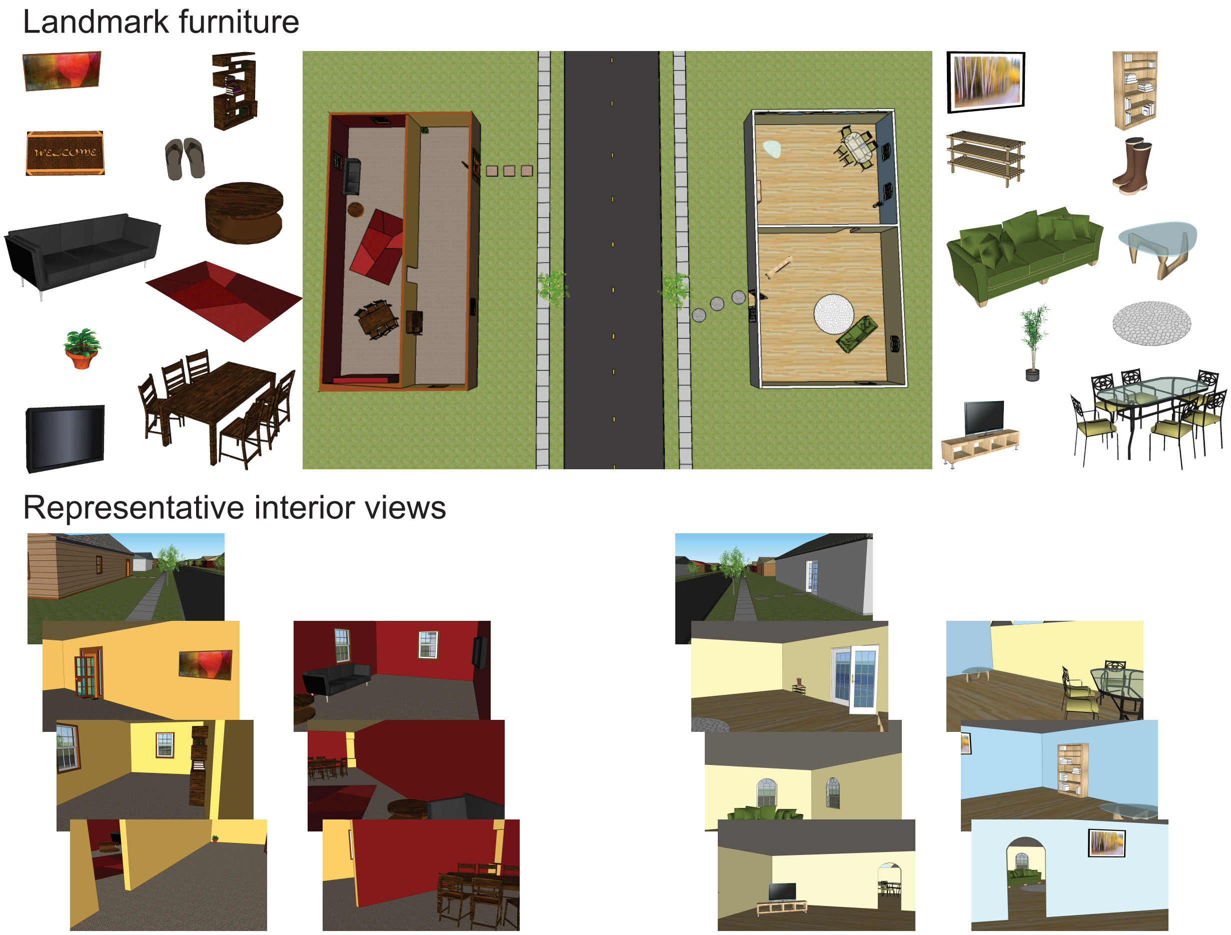

Supplementary Figure 1. Birdseye view of the houses showing layouts, landmark furniture, and representative interior views devoid of studied objects.
This figure is not included under the Creative Commons licence for the article; all rights reserved.

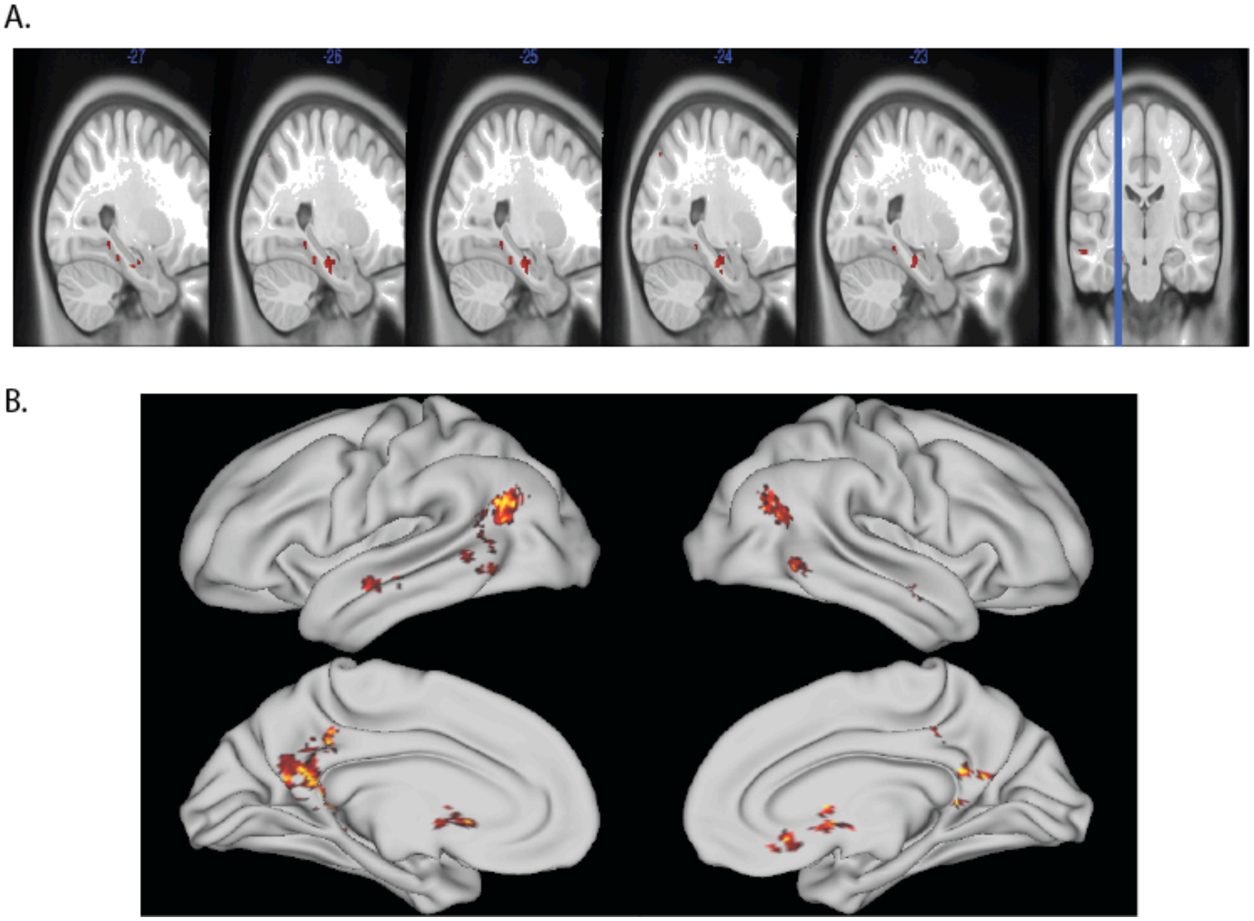

Supplementary Figure 2. Recollection-related activity in the hippocampus and neocortex. Results from the Univariate analysis contrasting Recollection trials against Familiar hit and Miss trials (FWE $p < 0.05$) are shown for (A.) the hippocampus on a T1-weighted template brain, and (B.) for the neocortex, rendered on an inflated template brain from Workbench (HCP S900 template).

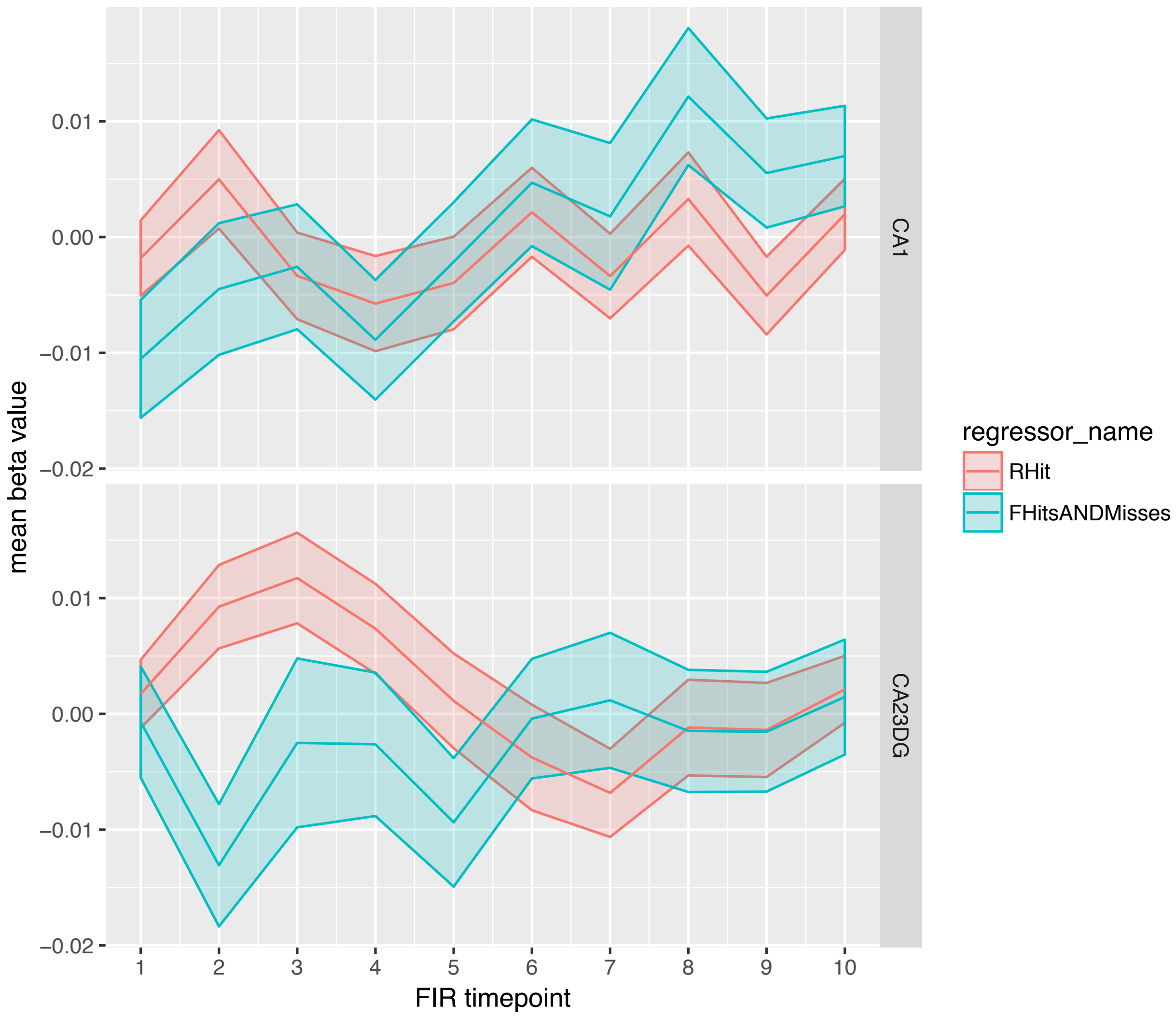

Supplementary Figure 3. Estimated finite impulse responses (FIR) for each subfield delineated within body of hippocampus and restricted to left hemisphere. Each FIR timepoint represents estimated activity at a single TR (2.01 seconds). Responses are shown separately for remembered hits (red) and familiar hits and misses (teal).

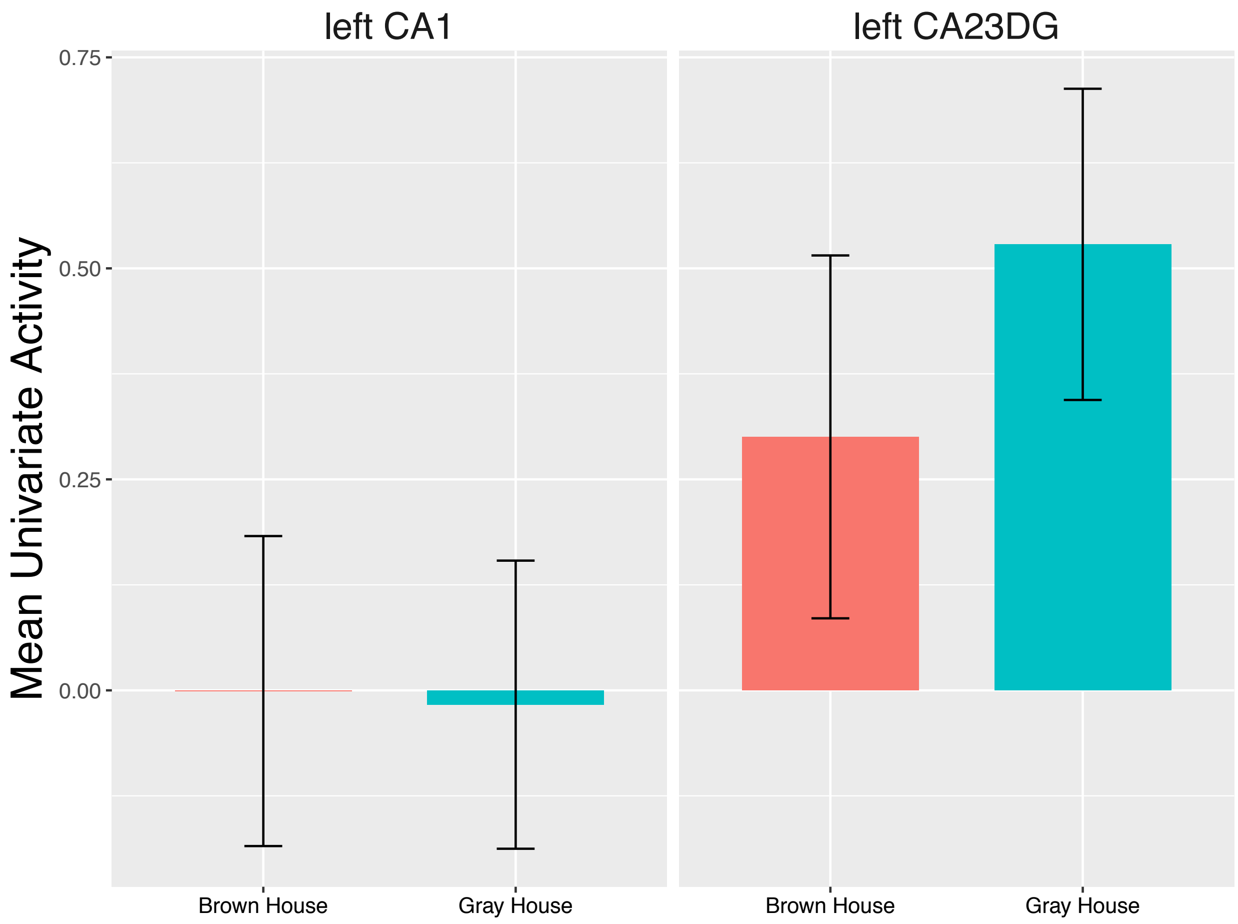

Supplementary Figure 4. Mean univariate activity for each subfield defined within the body of hippocampus restricted to left hemisphere for each spatial context (house). Subfield-specific univariate activity was estimated for recollected objects within each spatial context. Neither subfield reliably differed in its activity between the two spatial contexts (CA1: $F(1, 22) = 0.92$, $p = 0.92$; CA23DG: $F(1, 22) = 3.17$, $p = 0.09$).

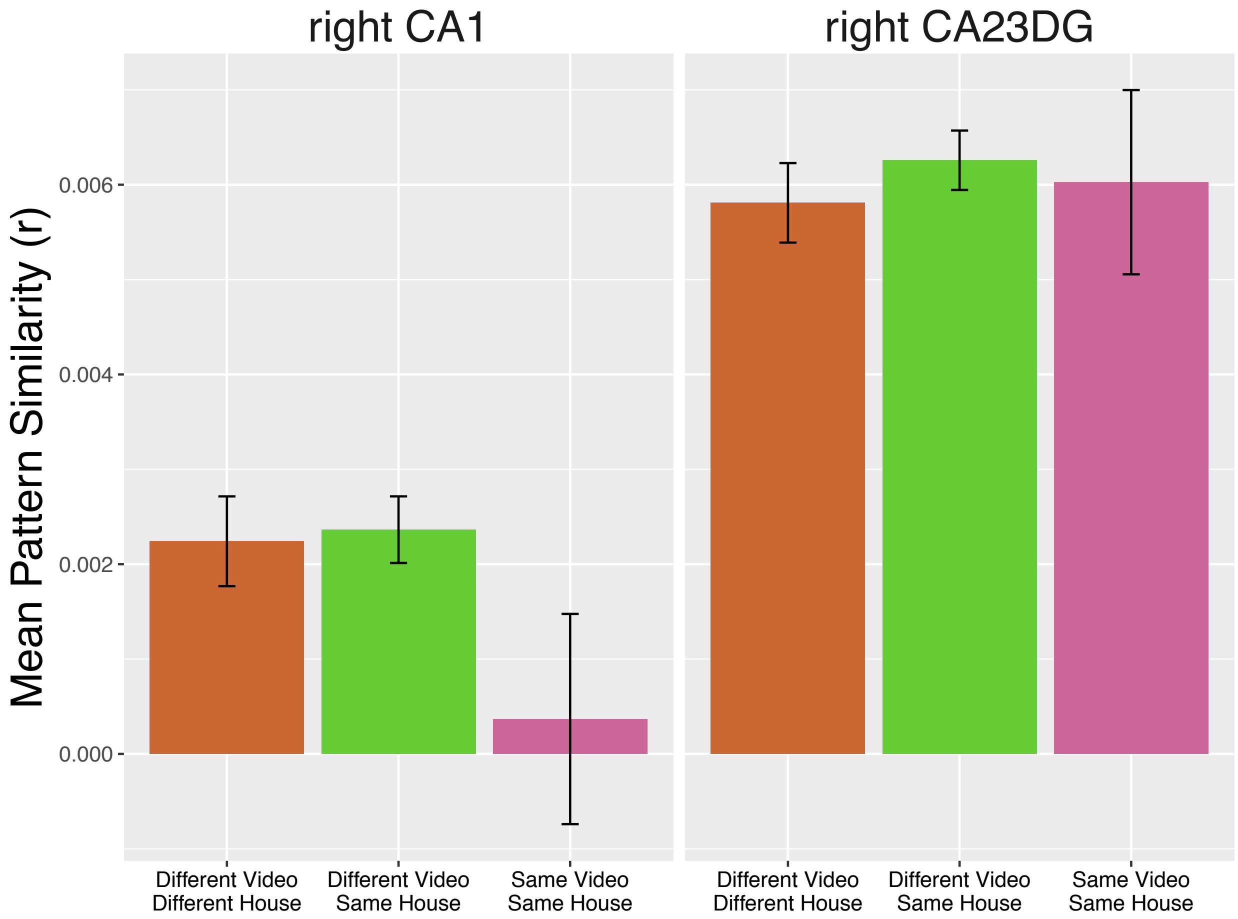

Supplementary Figure 5. Neural pattern similarity for spatial and episodic contexts in right hemisphere. There were no reliable differences in coding across CA1 and CA23DG for items on the basis of spatial ($\chi^2(2) = 0.19$, $p = 0.66$) or episodic context ($\chi^2(2) = 1.23$, $p = 0.27$).

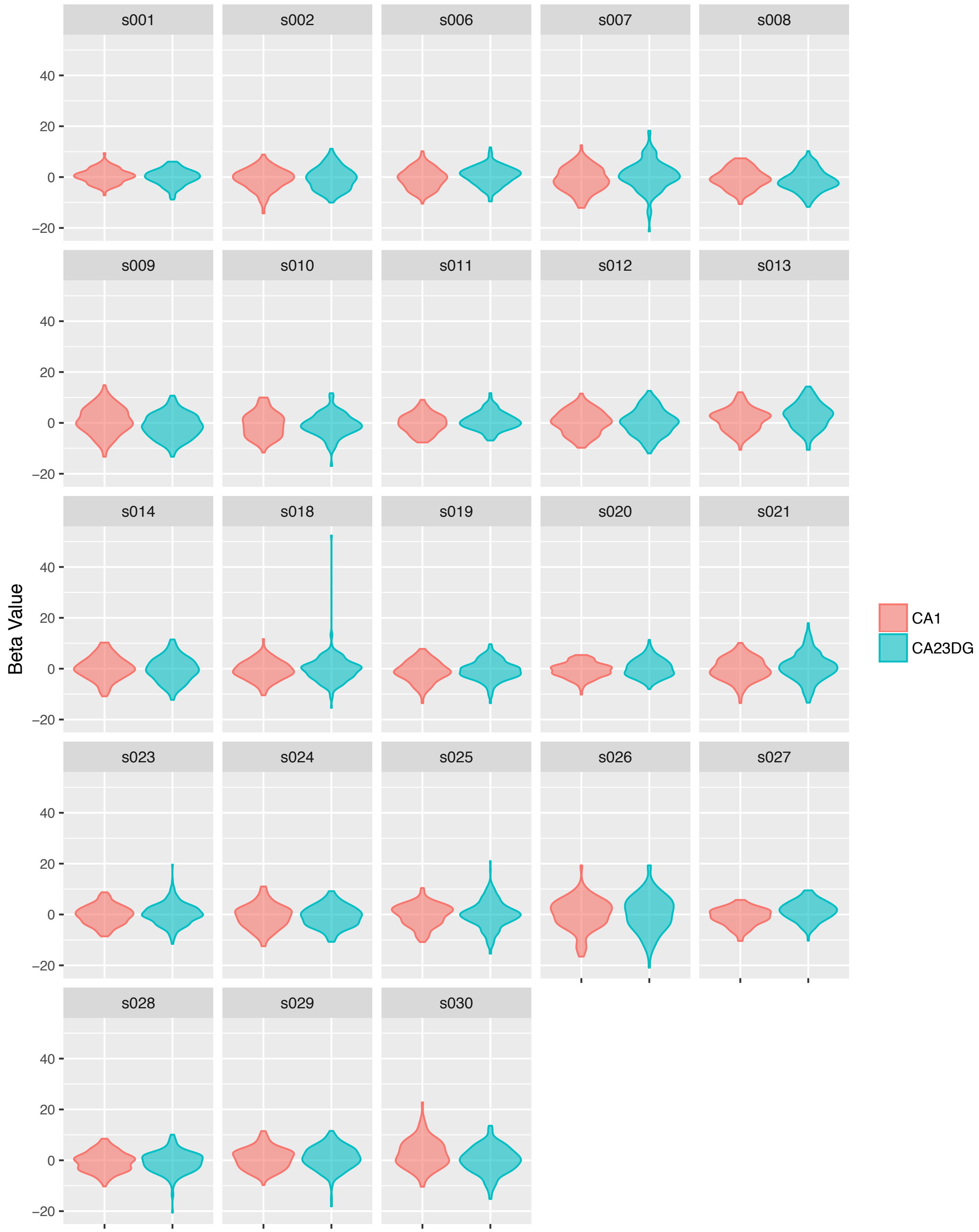

Supplementary Figure 6. Distribution of beta values for each subfield in left hemisphere by participant. Beta distributions were highly overlapping with no difference in either the mean (t(22) = -1.14, *p* = 0.26, d = -0.24) nor median (t(22) = -1.67, *p* = 0.11, d = -0.32) beta value between ROIs. Removal of s018 did not change the pattern of results.

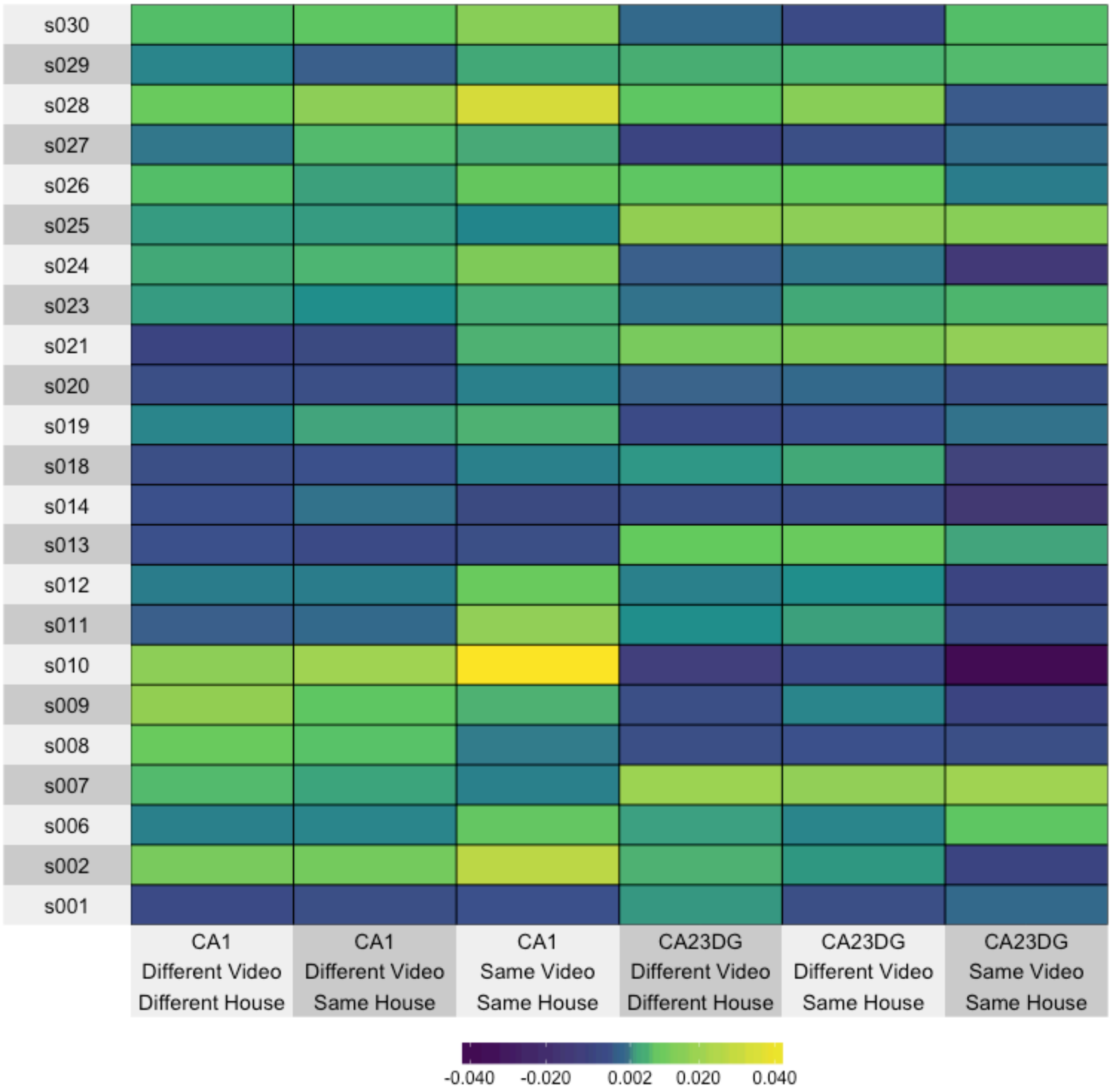

Supplementary Figure 7. Mean pattern similarity values for each subject (rows) broken down by ROI and condition (columns).

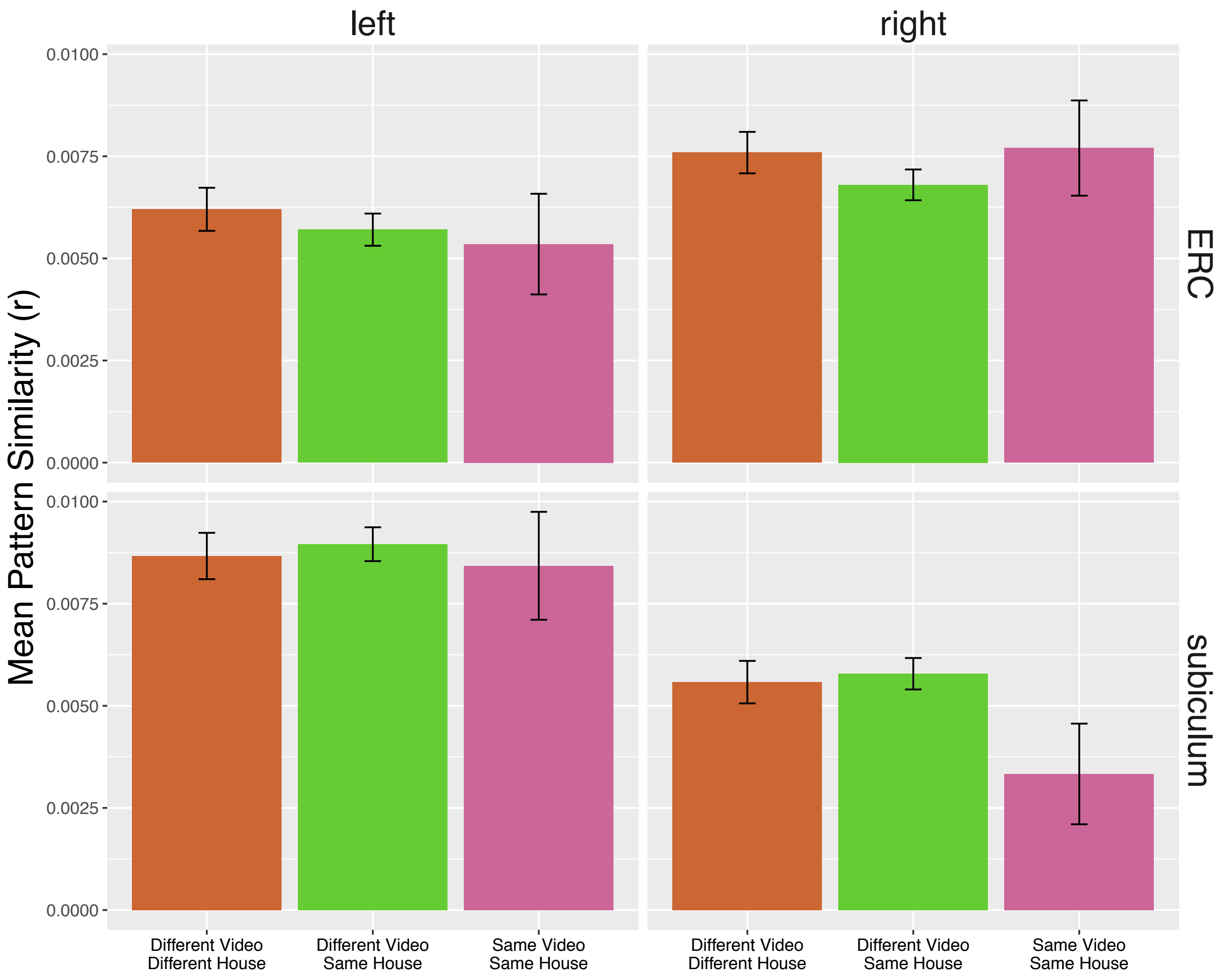

Supplementary Figure 8. Neural pattern similarity for spatial and episodic contexts in subiculum and entorhinal cortex (ERC). Neither subfield reliably differed in its representation for items studied in the same video (Same Video Same House) versus for items in different videos (Different Video Same House) nor for items based on their spatial context similarity (Different Video Different House vs. Different Video Same House).

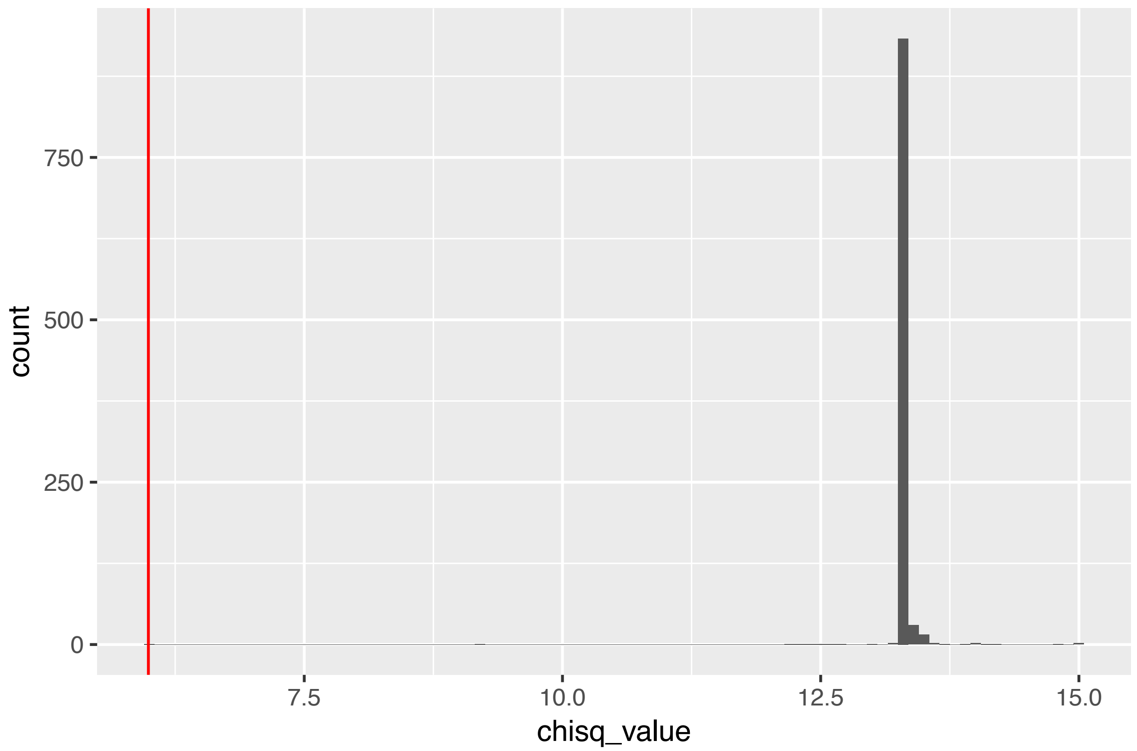

Supplementary Figure 9. Results from analyses using equal numbers of trial pairs. To ensure that our results could not merely be explained by the number of trials in each condition, we took the condition with the smallest number of trial pairs for each subject and randomly re-sampled the other conditions to have this reduced number of trial pairs. We then ran a mixed effects model to test for a significant ROI x Context Similarity x Hemisphere interaction. This procedure was repeated 1,000 times. Observed chi-square values from the 1,000 iterations are plotted as well as a line (red) showing the threshold for significance at $p < .05$. Across all of these random samples of trials, we obtained a significant result.

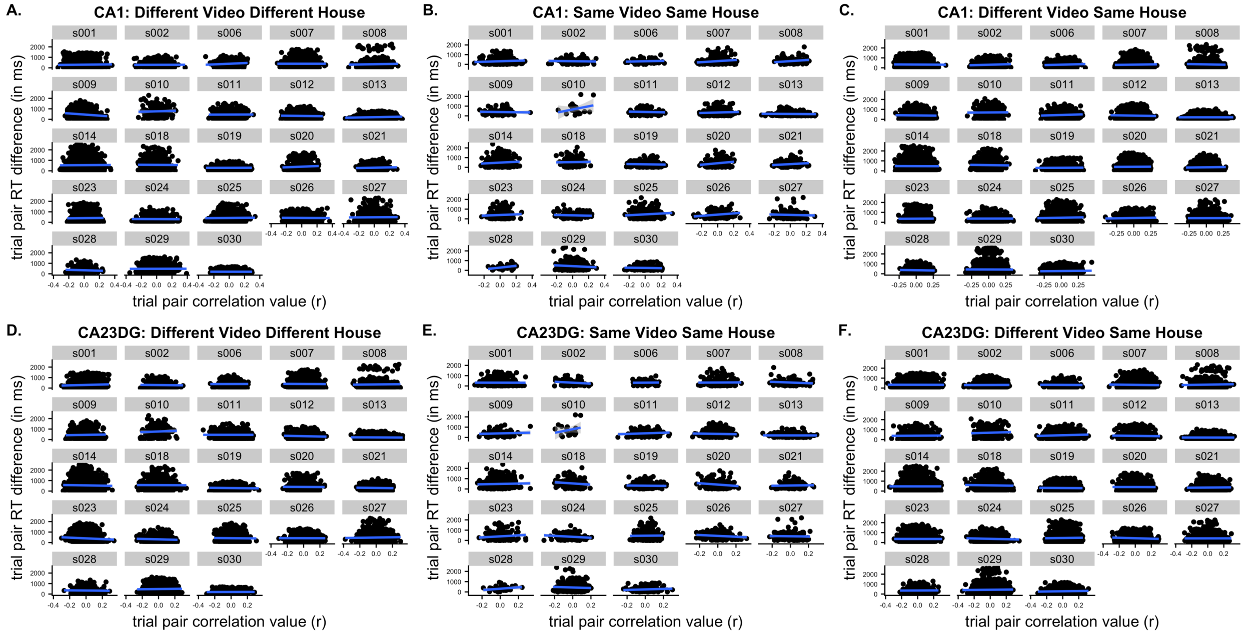

Supplementary Figure 10. Correlations between differences in reaction time between pattern similarity trial pairs and pattern similarity values with linear fit lines (blue). Plots for left CA1 (A-C) and CA23DG (D-F) show no reliable relationship between the difference in reaction times and the pattern similarity value for trial pairs. Including a random effect of reaction time difference in the mixed models did not change the results reported in the main analyses: There was a significant ROI x Context Similarity x Hemisphere interaction (χ^2 (2) = 13.31, p = 0.001). Follow up analyses revealed that this was driven by a reliable interaction between ROI and Context Similarity in left hemisphere (χ^2 (2) = 15.65, p < .001). When assessing the representation of Context Similarity in our left hemisphere ROIs, we found no difference between the subfields on the basis of Spatial Context Similarity (χ^2 (1) = 0.096, p = 0.78) whereas we did see a significant interaction indicating differences in representation for Episodic Context Similarity (χ^2 (1) = 15.47, p < .001). Removing s010 also did not change any observed effects.

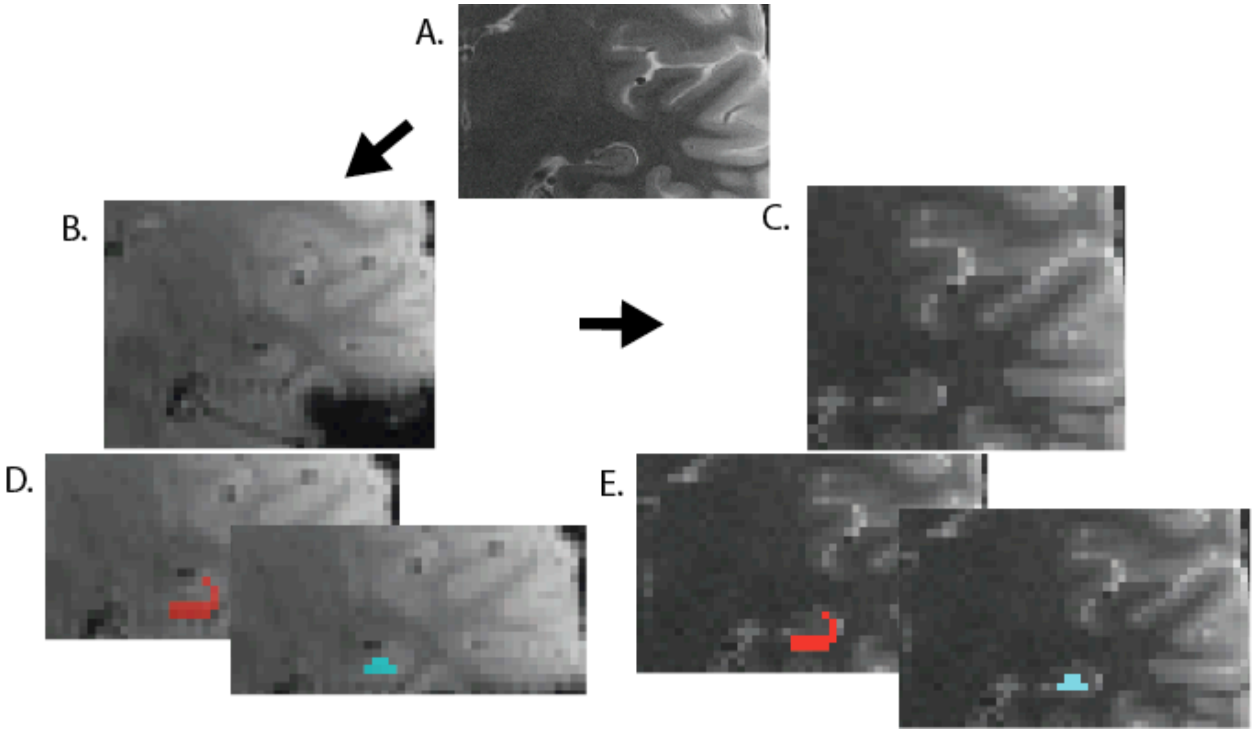

Supplementary Figure 11. Registration of ROIs to EPI data. T2 images in high-resolution (0.4 x 0.4 x 1.9 mm voxels) native space (A) were co-registered to the mean functional image (B). A resulting co-registered T2 image is shown in panel C. These parameters were then use to reslice the native-space ROIs that had been defined in T2 space into functional space (1.5 mm isotropic voxels). Co-registered ROIs of interest (CA1/red, CA23DG/blue) are shown overlaid on the mean functional image (D) and co-registered T2 image (E) for a representative subject's left hemisphere.

Supplementary Table 1. Memory performance for object recognition (scanned) and spatial context source memory (unscanned). Reaction time differences (in milliseconds) for conditions of interest in pattern similarity (PS) analyses (scanned).

Item Recognition	
Correct Responses	Mean response rate +/- SD
"R" / old items	0.68 +/- 0.17
"F" / old items	0.25 +/- 0.15
"N" / new items	0.90 +/- 0.09
Incorrect Responses	
"R" / new items	0.01 +/- 0.02
"F" / new items	0.09 +/- 0.02
"N" / old items	0.07 +/- 0.04

Item Recognition by PS condition	Mean RT difference (ms) +/- SD
sameVideo_sameHouse trial pairs	0 +/- 511.94
differentVideo_sameHouse trial pairs	0 +/- 512.28
differentVideo_differentHouse trial pairs	-4.59 +/- 514.85

Spatial Context Source Memory	
Response Type	Mean response rate +/- SD
"old" / old item	0.71 +/- 0.11
"new" / old item	0.26 +/- 0.10

Supplementary Table 2. Coordinates (in Montreal Neurological space), region labels (from Harvard Oxford Probablistic Atlases in MRICron), and cluster size (k), and t-values for the univariate contrast of recollection hit trials as compared to the combined activity for familiarity hits and misses (FWE corrected, p <.05). Clusters include local maxima as well as separate clusters >8mm apart as reported by SPM8.

MNI x	MNI y	MNI z	Region	Hemi	cluster size (k)	t
24	-39	-15	Fusiform	R	108	7.92
32	-40	-9	Lingual gyrus	R	-	4.69
40	-73	34	Lateral occipital cortex	R	1517	7.71
46	-72	28	Lateral occipital cortex	R	-	7.4
44	-63	30	Lateral occipital cortex	R	-	6.6
-10	-58	22	Retrosplenial cortex	L	2134	7.62
12	-57	19	Retrosplenial cortex	R	-	6.93
-6	-45	39	Retrosplenial cortex	L	-	6.84
-42	-67	28	Lateral occipital cortex	L	2668	7.37
-39	-78	36	Lateral occipital cortex	L	-	6.87
-44	-55	19	Angular gyrus	L	-	6.83
-63	-9	-18	Middle temporal gyrus	L	383	7.3
-50	-15	-20	Middle temporal gyrus	L	-	6.05
-56	-16	-12	Middle temporal gyrus	L	-	5.28
6	-48	3	Posterior cingulate	R	125	6.6
4	-54	9	Precuneus	R	-	5.54
-24	-21	-20	Hippocampus	L	88	6.37
3	12	-8	Subcallosal cortex	R	254	6.28
-4	18	-5	Subcallosal cortex	L	-	5.89
0	12	1	Subcallosal cortex	-	-	5.52
-2	-49	60	Precuneus	L	210	6.07
9	-40	39	Posterior cingulate gyrus	R	-	5.08
4	-37	45	Posterior cingulate gyrus	R	-	4.48
-32	-36	-15	Fusiform	L	92	5.68
-26	-42	-9	Lingual gyrus	L	-	4.27
-18	-37	-12	Hippocampus	L	-	3.85
6	27	-11	Subcallosal cortex	R	133	5.68
3	35	-15	Frontal medial cortex	R	-	5.12
62	-57	-9	Middle temporal gyrus	R	111	5.5
64	0	-21	Middle temporal gyrus	R	89	4.88
58	-9	-24	Middle temporal gyrus	R	-	4.84
68	-7	-21	Middle temporal gyrus	R	-	4.78
